# Clinical outcomes and return to sport after single-stage revision anterior cruciate ligament reconstruction by bone-patellar tendon autograft combined with lateral extra-articular tenodesis

**DOI:** 10.1007/s00590-022-03352-6

**Published:** 2022-08-18

**Authors:** Luigi Zanna, Giabbani Niccolò, Innocenti Matteo, Joseph Malone, Civinini Roberto, Matassi Fabrizio

**Affiliations:** 1 University of Florence, Orthopaedic Clinic CTO, Largo Palagi 1, 50139 Florence, Italy; 2 Department of Respiratory Medicine, Medical Clinic, Ullevål Hospital, Oslo University Hospital, Oslo, Norway

**Keywords:** Anterior cruciate ligament, Revision ACLR, Single-stage revision, Bone patellar tendon bone, Lateral extra-articular tenodesis

## Abstract

**Purpose:**

The anterior cruciate ligament reconstruction (ACLR) failure rate continues to increase. Involvement of a young population with a desire to return to sport, explains the increased need for ACLR (revACLR) revision. The aim of this study was to evaluate clinical outcome, complications, failure rate and return to sport of a single-stage revACLR using bone patellar tendon-bone (BTBT) combined with lateral extra-articular tenodesis (LET).

**Material And Methods:**

A retrospective analysis was performed on 36 patients who underwent revACLR. Knee stability was assessed by Lachman and Pivot shift test. Objective anterior laxity was determined by KT-2000 arthrometer. The IKDC subjective, Lysholm, ACL-RSI Scores, level of sport activity and Forgotten Joint Score-12 were recorded.

**Results:**

Of 36 patients, we collected data from 17 who underwent single-stage revACLR with autologous BTBT combined with LET, performed using an extra-articular MacIntosh procedure as modified by Arnold–Coker. The side-to-side difference in Lachman test and Pivot shift test significantly improved postoperatively. The subjective IKDC, Lysholm and ACL-RSI significantly improved from 71.4 ± 9.03 to 92 ± 6.9, from 58.3 ± 19.3 to 66.8 ± 27.7 and from 50.4 ± 12.2 to 68.6 ± 24.5, respectively during the post-operative follow-up. Ten patients (58.8%) returned to their desired level of sport. One patient was considered a failure because of the postoperative laxity.

**Conclusion:**

Single-stage revACLR with BPTB combined with LET is a safe procedure that shows good objective and subjective outcomes, and a high rate of return to the same level of sport. Reducing rotational instability and strain on intra-articular reconstructed structures results in a low rate of complications and failure.

## Introduction

Over the last decade, the anterior cruciate ligament reconstruction (ACLR) has become an increasingly common orthopedic procedure, for the growing number of injuries in pivoting sports, other athletic activities and not only [1–3]. Despite the improvements in surgical techniques, the ACLR failure rate continues to increase, ranging between 0 and 25% [4, 5]. This is largely due to the involvement of a young active population [6, 7] with the desire to return to sport at the pre-injury level leading to an increase in the indication for operative revision ACL reconstruction (revACLR), especially in athletes. Historically, revACLR was often performed as a two-stage procedure, dealing with tunnel malposition and widening. The window of time of 3–6 months, between two stages [8], exposes the knee to instability and meniscal and chondral injuries [9]. In order to avoid the drawbacks of a delayed reconstruction, the interest in single-stage revision showed significant improvements in patient function and comparable results in terms of graft failure and outcomes [10]. Furthermore, the interest in knee Anterolateral Ligament (ALL) has recently increased [11] for the biomechanical role of the ALL in controlling rotational laxity, internal rotation and pivot-shift [11, 12]. Some authors highlighted how an isolated intra-articular ACLR did not fully restore normal knee joint kinematics and tibial rotation, resulting in residual rotational instability with an increased risk of failure [13]. In the recent literature, a consensus that provides the main indication for ALL reconstruction in the context of revACLR surgery has not been described yet and only a few case series are reported [13]. The main purpose of the present study was to evaluate the clinical outcomes, and the complication and failure rates of a single-stage revACLR using the bone patellar tendon-bone (BPTB) in conjunction with a lateral extra-articular tenodesis (LET). The hypothesis was that the combined procedures are safe and produce good clinical results, especially in terms of residual rotatory laxity, failure rate and return to sport.

## Materials and methods

**Fig. 1:**
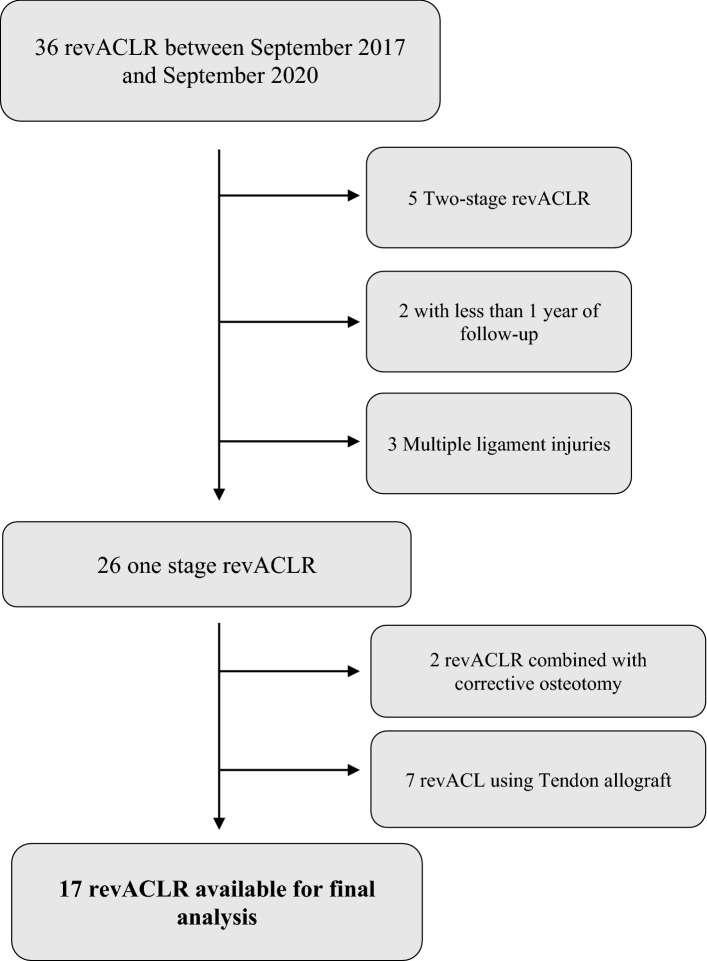
Patients excluded and included in the study

We retrospectively reviewed 36 patients who underwent revACLR in our Institute by a single surgeon between September 2017 and September 2020. Inclusion criteria were single-stage revACLR with ipsilateral BPTB autografts and LET, no other ligaments’ reconstruction and/or coronal malalignments that require correction osteotomies, and a minimum of 12 months of follow-up (FU). We excluded patients with multiple ligament injuries, including high-grade partial ruptures of the medial collateral ligament and posterolateral complex, that could affect the pivot shift, patients with contralateral ACL tear or surgery, patients treated with two-stage reconstruction, not eligible for one-stage surgery, and with a FU lower than 12 months (*x* = 19) Fig. 1. Demographic data was collected. A clinical evaluation of ligamentous knee stability was assessed by Lachman and Pivot Shift tests. Objective anterior laxity was determined with the KT-2000 arthrometer (MEDmetric, San Diego, CA). The side-to-side difference in anterior displacement at the maximal manual force was calculated and recorded before surgery and at final FU by two expert orthopedic surgeons. The International Knee Documentation Committee (IKDC) subjective scores, Lysholm score, ACL-RSI and level of sport activity were recorded pre-operatively and at the last FU. Furthermore, we evaluated the Forgotten Joint Score-12 (FJS-12) of the knee. Pre-operative radiograph, MRI, and Computed Tomography (CT-scan) were obtained. The first FU evaluation was performed 15 days after surgery. Then we evaluated each patient at 1, 3, 6 months and 1 year after surgery.

### Preoperative planning

A CT scan was obtained preoperatively to evaluate position and diameter of tibial and femoral tunnels [14]. A 3D-CT reconstruction provided the femoral lateral condyle in the sagittal view (with suppression of the medial femoral condyle) and of the tibial plateau in the axial view. With a standard axial, sagittal and coronal CT scan we assessed the diameter and the anteroposterior and mediolateral location of the tibial and femoral tunnels [15, 16]. Bernard et al. grid [17] was superimposed on 3D-CT reconstruction of the medial articular surface of the lateral femoral and of the articular surface of the tibial plateau.

### Femoral tunnel

**Fig. 2:**
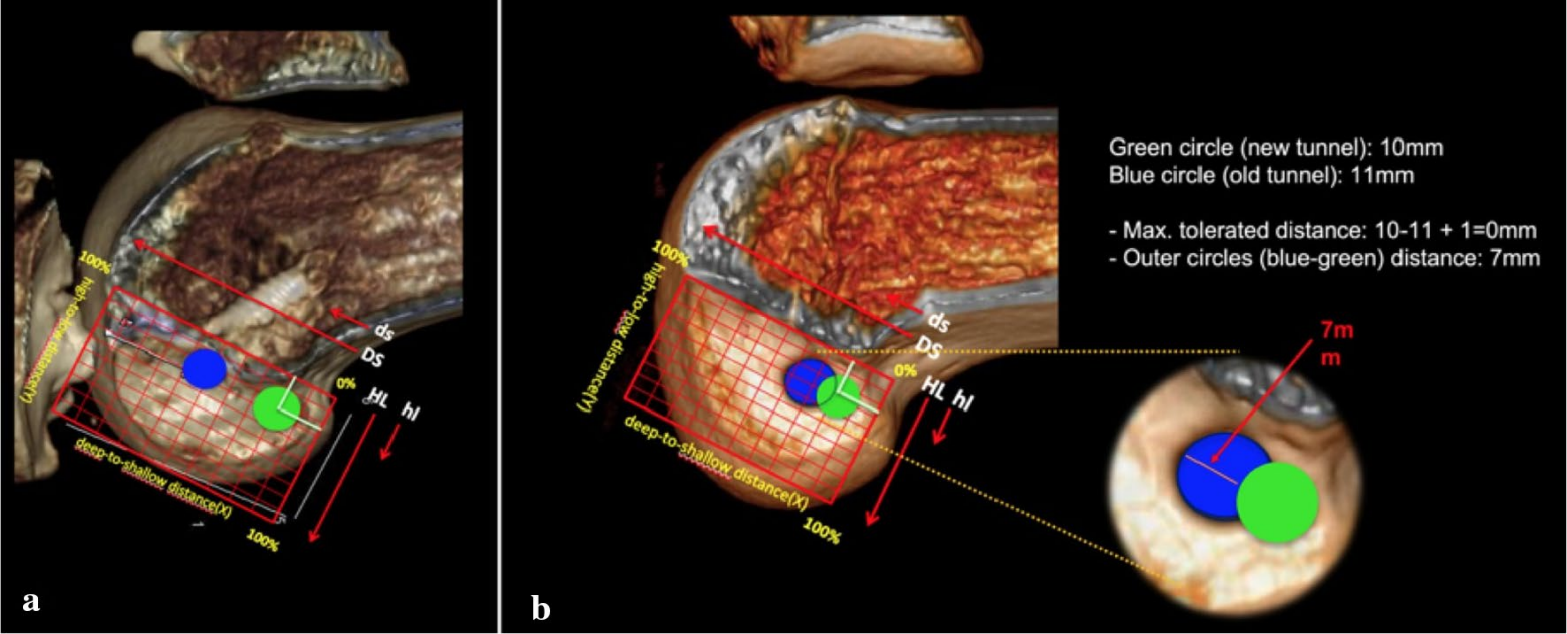
**A** 3D-CT reconstruction of femoral lateral condyle in the sagittal view (with suppression of the medial femoral condyle), with no superimposition between the previous (blu) and the new planned tunnels (green) **B** 3D-CT reconstruction of femoral lateral condyle in the sagittal view, case of not tolerated superimposition between the old and new planned tunnels, that required two-stage revACLR (colour figure online)

The location of the previous femoral tunnel and anatomical center of the ACL femoral footprint was identified. The distance between the outer diameters of the two tunnels was measured. If we did not find any superimposition we proceeded with a single-stage revACLR (Fig. 2a). In the case of a superimposition between the old and new planned tunnels we tolerated a distance between the outer circle of the two tunnels equal to the difference between both tunnels diameter plus maximum 1 mm more (Fig. 2b). In case the new planned 10 mm tunnel could not completely cover the previous tunnel diameter, a two-stage procedure was performed.

### Tibial tunnel

**Fig. 3:**
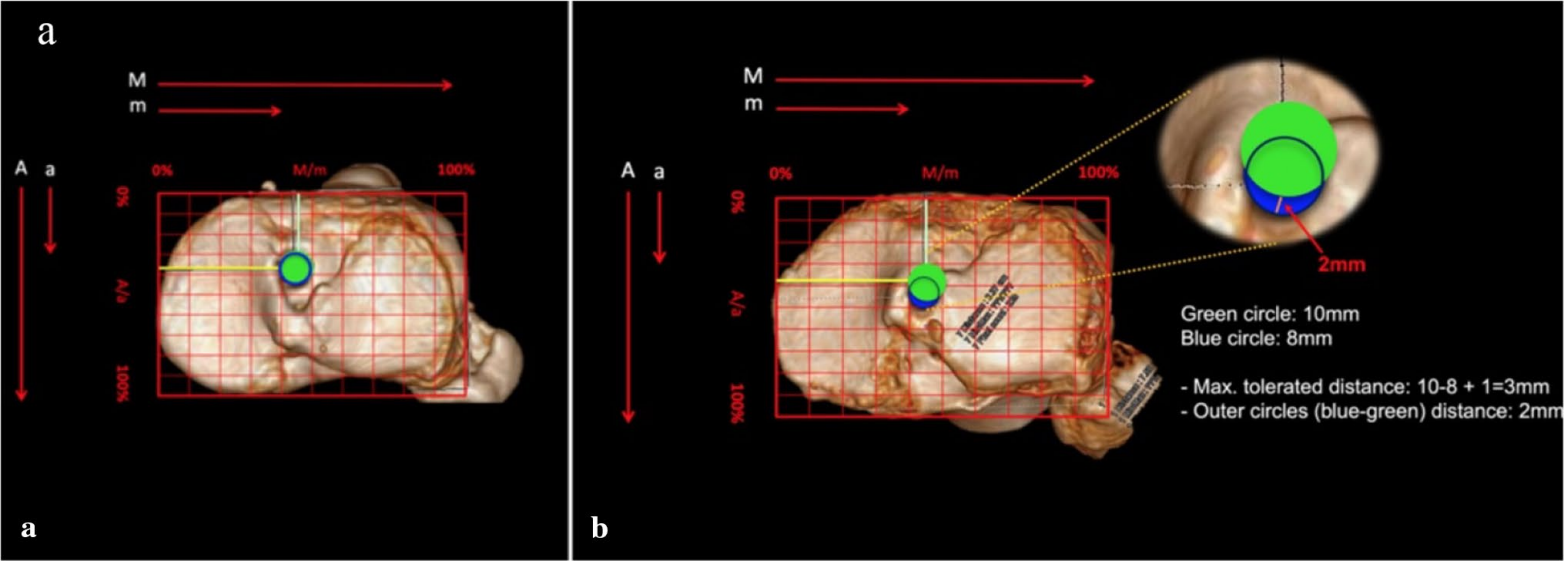
**A** 3D-CT reconstruction of the tibial plateau in the axial view with complete superimposition between the previous (blu) and the new planned tunnels (green); **B** 3D-CT reconstruction of the tibial plateau in the axial view with tolerated superimposition, candidate for single-stage revACLR (colour figure online)

The previous tibial tunnel and the anatomical center of ACL tibial footprint were identified. The distance between the outer diameters of the two tunnels was measured. In cases of anatomical tunnel positioning (Fig. 3a) or no superimposition of the new planned tunnel with the old one, we performed a single-stage revACLR. In cases of overlapping tunnels (Fig. 3b), when the distance between them was more than the difference between the diameter of both tunnels plus maximum 1 or more millimeters a two-stage procedure was required.

### Surgical technique

**Fig. 4:**
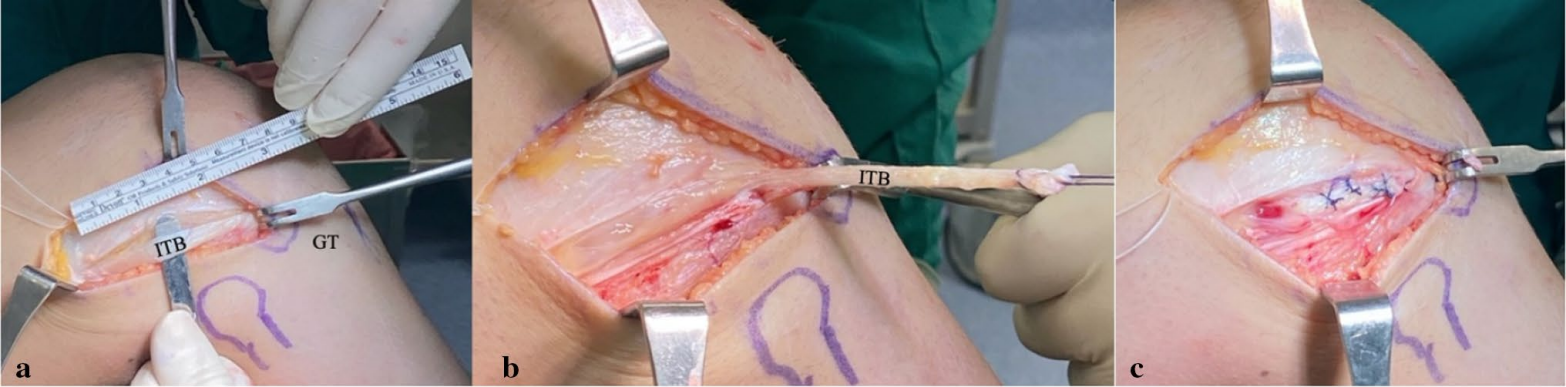
**A** Ileotibial band (ITB) incision starting from the Gerdy’s tubercle (GT) for 10 cm proximally **B** The harvested ileotibial band before passing under the lateral collateral ligament **C** The ileotibial band sutured to the lateral collateral ligament and to itself and to Gerdy’s tubercle

After spinal anesthesia, patients were positioned supine on the operating table with an inflated tourniquet at the upper thigh. The central one-third of the ipsilateral patellar tendon, 10 mm wide, was harvested and then prepared on the Arthrex preparation station. A diagnostic arthroscopy was initially performed. According to type, meniscal lesions were treated by either partial meniscectomy or repair. The tibial marking hook drill guide (Arthrex) angle was set according to the pre-operative planning, and the tip of this guide was placed into the ideal center of ACL tibial insertion. Through a small incision in the proximal anteromedial tibia, a guide pin was introduced, and a tibial tunnel was created using a cannulated drill with a diameter correspondent to the graft (10 mm). The femoral tunnel was performed using an outside-in footprint femoral ACL guide (Arthrex) placed in the AL portal, with the tip of the guide centered on the ideal femoral ACL footprint and based on pre-operative CT-scan planning. A femoral tunnel was then created using a cannulated drill matching the graft’s diameter. The BPBT graft was passed through the femoral and tibial tunnels and fixed to the femur with an interference screw. A second interference screw was used for fixing the graft on tibial side, at 30° of knee flexion. LET was performed after ACL fixation via a MacIntosh modified Arnold–Coker technique. The lateral epicondyle, fibular head and Gerdy's tubercle were identified. An 8 cm skin incision was made starting from the lateral epicondyle in the direction of the Gerdy's tubercle. The iliotibial band (ITB) was identified and a 1 cm wide and 10 cm long tape was incised and detached proximally, preserving the distal insertion (Fig. 4a–b). The released portion of the ITB was passed under the lateral collateral ligament in anterior to posterior direction. The ITB strip was then turned on itself and sutured under tension with periosteal stitches to Gerdy’s tubercle, at 90° of knee flexion with tibia in neutral rotation (Fig. 4c). The ileotibial tract was also sutured to the LCL for additional stability.

### Post-operative rehabilitation

All patients followed the same post-operative rehabilitation protocol. The knee brace was not used, and patients were allowed to weight bear on the operated leg and to recover full range of motion (ROM) from the first postoperative day. When a meniscal suture was performed, the postoperative protocol included a restriction of weight bearing for 4 weeks and the use of a knee brace with a maximum of 90° of flexion. Closed kinetic chain quadriceps strengthening exercises and progressive exercises to recover ROM were encouraged from the 1st postoperative day, with the aim to obtain a ROM of 0–90° within the first 30 days. Straight-line jogging and open kinetic chain exercises were allowed after 3 months. Return to full sport activity was allowed, depending on the type of sport, between 6 and 9 months postoperatively.

### Statistical analysis

Statistical analysis was performed using SPSS ver. 25.0 (SPSS Inc., Chicago, IL, USA). Categorical variables are presented as numbers and percentages and continuous variables as the mean and standard deviation. Shapiro test was used to assess normality of distribution. Continuous variables were compared using paired and unpaired t-test, continuous and ordinal variables with Wilcoxon test as appropriate. A two-sided *p*-value < 0.05 was defined to be considered statistically significant.

## Results

**Fig. 5:**
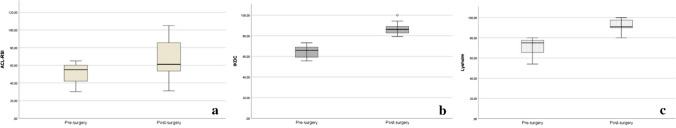
**A** ACL-RSI score range pre and post-surgery, **B** IKDC score range pre and post-surgery, **C** Lysholm score range pre and post-surgery. The box height represents the interquartile range (*Q*1–*Q*3), the line within the box is the median value, the lower and upper whiskers represent the lowest and the highest samples, respectively. Circles in boxplots represent outlier samples (> 1.5xIQR)

Of 36 patients, we retrospectively reviewed 17 patients who underwent one-stage revACLR with autologous BTBT, combined with LET. Fourteen were male (82.3%) and 3 female (16.7%). The mean age was 26.4 ± 6.3. The right side was involved in 12 patients (70.6%) and left side in 5 (29.4%). The FU was 29 ± 10.9 months, and the median hospital stay was 1 day (range 1–2). Meniscal lesions were diagnosed in 10 patients (58.8%): 4 underwent medial meniscus repair, 3 lateral meniscus repair and 3 lateral meniscectomy. Results from subjective and objective measures are reported in Table 1 and Fig. 5a, b and c. Ten patients (58.8%) returned to their desired type and level of sport, 3 (17.7%) to same activity, but at a lower level. Three patients (17.7%) preferred a less stressful activity for reasons other than the result of the operation (work, family, and responsibilities) and 1 did not return to sport activity (5.8%) due to residual rotational laxity. Patients who did not return to sport had a significant lower ACL-RSI score than who did *(p* = *0.009)*. The FJS-12, to assess the patient's awareness of their joint at final FU, showed high values (Median 92 and range 78–98). This score indicated less awareness of the joint and a higher level of the affected knee forgetting during activities of daily living. One patient was considered a failure because of the residual post-operative laxity on examination (KT-2000 reading > 5 mm side-to-side difference, Lachman 2 + , and pivot shift 2 +). One patient (5.8%), after a relevant contact trauma during sport, had a failure of the revACLR.

**Table 1:** Clinical and functional outcome pre- and post- revACLR combined with lateral extra-articular tenodesis

	Preoperative	Postoperative	*P* value
*Lachmann*			
< 3	0	11	*p* = 0.003
3–5	0	5	
6–10	8	1	
> 10	9	0	
*Pivot shift*			
Negative (0)	0	11	*p* = 0.003
Glide (1 +)	0	5	
Clunk (2 +)	6	1	
Subluxation (3 +)	11	0	
Lysholm score	71.4 ± 9.03	92 ± 6.9	*p* = 0.003
IKDC subjective score	58.3 ± 19.3	66.8 ± 27.7	*p* = 0.0001
ACL-RSI scale	50.4 ± 12.2	68.6 ± 24.5	*p* = 0.021
VAS scale		2.8 ± 1.9

## Discussion

Surgery for revACLR is technically more demanding than primary reconstruction with a higher rate of failure [18]. Some authors [19] report the importance of performing adjunctive ALL extra-articular procedures to decrease rotational laxity, reducing the failure rate. To our best knowledge, few authors reported single-stage revACLR with autologous BPTB combined with LET [20]. The analysis of our cohort of patients reported significant improvement in both subjective and objective postoperative outcomes, with high rate of return to the same level of sport (76.5%). The postoperative mean Lysholm of our cohort was 92 ± 6.9, comparable to Grassi et al. [20] that studied revACLR surgery combined with lateral extra-articular procedures. The authors [20] analyzed the Lysholm score of 630 patients, reporting an average of 88.9 points at final FU. Conversely, the mean postoperative IKDC scores of our patients (66.8 ± 27.7) resulted slightly lower than other reports [5, 19]. Despite overlapping results of Lysolm score, authors such as Redler [5] and Mazzola [19], that performed the same surgical procedure, reported higher results of IKDC subjective score, 85.7 ± 12.3 and 88.4 respectively. Otherwise, we registered better both Lysholm and IKDC scores than authors that performed different lateral tenodesis procedure [21]. Our clinical scores were comparable to studies that analyzed isolated revACLR [22, 23]. Glogovac et al. [24], in their systematic review, analyzing 13 studies regarding isolated revACLR, reported average of IKDC subjective and Lysholm scores postoperatively between 43 and 86.1 and 83.8 to 90.5 respectively. In our cohort, the addition of LET was effective in reducing rotational laxity. Significant improvement in the rotational stability and a low rate (5.8%) of abnormal or severely abnormal postoperative pivot-shift (2 + /3 +) were registered, with results similar to primary ACL reconstruction (2%) [25]. Our high postoperative clinical outcomes and optimal recovery of rotational stability are confirmed in the literature [26, 27]. Lee et al. [26], in the MARS cohort, reported significantly better clinical results and reduction in residual pivot shift in the group that underwent anatomic ALL reconstruction plus revACLR than the isolated revACLR. Other authors [27] reported a higher rate of negative pivot shift in patients with lateral tenodesis without influence on clinical scores. Postoperative complications included wound hematoma, implant removal due to local pain, peroneal nerve palsy, stiffness requiring arthroscopic arthrolysis, superficial infection and muscular hernia in the lateral approach, ranging between 0 to 16% [5, 10, 11, 21]. Our populations did not develop any major complication, confirming the safety of combining LET with intra-articular revACLR. Recent literature concerning revACLR failure revealed a rate ranging from 8 to 25%, with an increased incidence in the young population [5] and a higher failure rate in the presence of residual rotatory laxity [25]. Our failure rate is in line with the data in the literature [5, 25], one patient (5.8%) was considered failure due to residual post-operative laxity. Furthermore, we registered 1 case of instability after a relevant direct knee trauma during sport. Most of the patients who undergo revACLR surgery are young and active, with a desire to return to sport. Few studies evaluated both rate of return to sports and ACL-RSI scale after revACLR combined with LET procedure. Patients have to overcome the fear of further knee injury [28], and this psychological factor may explain why some of them with normal knee function does not recover the expected activity. In literature, a close correlation between self-confidence, motivation and return to sport was reported [22, 29]. The two most registered psychological tests are the ERAIQ (Emotional Responses of Athletes to Injury Questionnaire) and ACL-RSI. In our cohort we reported a significant postoperative improvement of ACL-RSI, furthermore, the subjects who returned to sports demonstrated better scores than those who did not *(P* = *0.009).* The objective rate of return to sport after revACLR has not been well described as for other less complex knee procedures [6, 30]. In our population, the 76.5% of our patients returned to sport, with more than 55% returning to the same pre-injury level. Similar results have been recently reported in the literature, with a rate of return to sport higher than 70%, with 40% of patients returning to the same pre-injury level [22, 24]. Despite the similar data, in our population, we registered 58.8% of patients that recovered to the previous competition level. This result is higher than large parts of studies that analyzed the activity after an isolated revACLR [22, 24]. The addition of the LET was associated with a greater rate of returning to the pre-injury sports level if compared with isolated revACLR, due to the significantly reduced rotational laxity [18, 18]. The aim of the surgeon, besides the restoring knee stability, is to provide a “normal” feeling in the affected knee. With this regard, we measured joint awareness using the FJS-12 reporting high values (Median 92 and range 78–98). Such good FJS-12 results may indicate a high degree of forgetting the joint in everyday life, according to good clinical results and high rate of return to sport related to the single-stage revACLR combined with LET. Our hypothesis was that a re-rupture after primary ACLR would result in more frequent ALL injuries and a positive high-grade pivot shift. Despite that most of the failures are related to surgical procedure technical, a residual rotatory laxity persists in a subgroup of patients. According to some authors [32] that reported a correlation between revACLR failures and persistent rotational instability during sports activity, we would suggest an additional LET after revACLR in patients with objective rotatory laxity.

The present study has several limitations. First, it is a nonrandomized and retrospective analysis, without a control group. Secondly, the FU was relatively short; longer FU may provide additional clinical results. Thirdly, knee rotational instability was assessed manually with the pivot-shift test by two surgeons, which is a subjective test. Finally, the sample of patients is relatively small. While the numbers are not ideal, they represent an important value if the short timeline is taken into consideration. Some positive aspects of this analysis are the standardized technique, always performed by the same surgeon, associated with homogeneous rehabilitation, which allows us to directly compare and validate results. Application of international scores (Lysholm, IKDC, ACL RSI and FJS-12) permits a direct comparison with different reports in the literature.

## Conclusion

The single-stage revACLR with BPTB combined with LET is a safe procedure that shows good objective and subjective clinical outcomes, and a high rate of return to the same level of sports activity. The significant reduction of rotational instability and of the strain on the intra-articular reconstructed structures results in a low rate of complication and ACLR failure and a “normal” feeling of the affected knee. According to our findings, we feel confident to recommend the association of LET to revACLR surgery. Further prospective studies with a larger cohort of patients are needed to support our results.

## Data Availability

All data are available in the main text and tables. Additional information can be provided if solicited.
